# Functional traits underlying performance variations in the overwintering of the cosmopolitan invasive plant water hyacinth (*Eichhornia crassipes*) under climate warming and water drawdown

**DOI:** 10.1002/ece3.9181

**Published:** 2022-08-04

**Authors:** Xiaolong Huang, Fan Ke, Qisheng Li, Yu Zhao, Baohua Guan, Kuanyi Li

**Affiliations:** ^1^ State Key Laboratory of Lake Science and Environment, Nanjing Institute of Geography and Limnology Chinese Academy of Sciences Nanjing China; ^2^ Sino‐Danish College University of Chinese Academy of Sciences Beijing China; ^3^ College of Environmental and Chemical Engineering Chongqing Three Gorges University Wanzhou China

**Keywords:** aquatic plants, biological invasion, climate warming, *Eichhornia crassipes*, functional traits, water drawdown

## Abstract

Reports of the Intergovernmental Panel on Climate Change (IPCC) indicate that temperature rise is still the general trend of the global climate in the 21st century. Invasive species may benefit from the increase in temperature, as climate can be viewed as a resource, and the increase in the available resources favors the invasibility of invasive species. This study aimed to assess the overwintering growth of the cosmopolitan invasive plant water hyacinth (*Eichhornia crassipes*) at its northern boundary. Using *E. crassipes* as a model plant, a cross‐year mesocosm experiment was conducted to determine 17 plant functional traits, including growth, morphological, root topological, photosynthetic, and stoichiometric traits, under climate warming (ambient, temperature rises of 1.5°C and 3.0°C), and water drawdown or water withdrawal (water depths of 1, 10, and 20 cm) treatments. The overwintering growth of *E. crassipes* was facilitated by climate warming and proper water drawdown, and climate warming played a leading role. A temperature rises of 3.0°C and a water depth of 10 cm were the most suitable conditions for the overwintering and rooting behavior of the plant. Controlling the temperature to within 1.5°C, an ambitious goal for China, still facilitated the overwintering of *E. crassipes*. With climate warming, the plant can overwinter successfully, which possibly assists it in producing and spreading new ramets in the vernal flood season. The new rooting behavior induced by ambient low temperature may be viewed as a unique growth adaptation strategy for a niche change, as it helps these plants invade empty niches left by dead free‐floating plants on the water surface following winter freezes. With continued global warming, the distribution of the plant may expand northward, and eradication of the plant during the winter water drawdown period may be a more effective strategy.

## Graphical abstract

A cross‐year mesocosm experiment was conducted using *Eichhornia crassipes* as a model plant under climate warming and water drawdown. The results revealed that the overwinter survival and growth of *E. crassipes* was facilitated by the two factors, and climate warming played a leading role. Controlling the temperature to within 1.5°C still facilitated the overwintering of the plant and eradication of the plant during winter water drawdown period may be more effective.

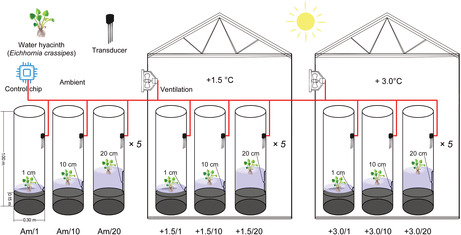

## 1 INTRODUCTION

The Intergovernmental Panel on Climate Change (IPCC) report indicates that in the centuries since the industrial revolution, the increase in anthropogenic greenhouse gas (GHG) emissions has accelerated, and the temperature rise is still the general trend of the global climate in the 21st century (IPCC, 2014; Stott et al., 2000). If the current trends continue, global warming will reach 1.5°C above preindustrial levels in the early 2030s, which is approximately 10 years earlier than the midpoint of the likely range (2030–2052), and even possibly 3.3–5.7°C by 2100 in high‐emission scenarios (IPCC, 2018, 2021). Changes in the average annual temperature, as well as the maximum and minimum temperatures, represent the influence of climate change on the ecosystem (Stachowicz et al., 2002). Extreme cold nights at high latitudes will warm up to 4.5°C with global warming of 1.5°C (IPCC, 2018). Global warming will change the distribution of plants, simplifying the food‐web structure and causing the extinction of endemic species, the loss of biodiversity, and the acceleration of biological invasion (Bellard et al., 2013; Clements & Ditommaso, 2011; Gallardo et al., 2016; García et al., 2018; Liu et al., 2017; O' Gorman et al., 2019; Ren et al., 2021). The assumption that global warming will promote plant invasions by increasing the overwintering potential of invasive species remains uncertain (Bradley et al., 2010; Early et al., 2016; Hulme, 2009; Seebens et al., 2015).

Biological invasions are a part of global change, and other aspects of global change have impacts on biological invasions (Dukes & Mooney, 1999; Feng et al., 2022). Biological invasions have been considered a major threat to global biological diversity and a cause of economic losses (Diagne et al., 2021; Dudgeon et al., 2006; Geist, 2011; Lu et al., 2013; Luque et al., 2014). Invasive species usually outperform native plants in terms of functional traits (Caplan & Yeakley, 2013; Kuebbing & Nuñez, 2016; Richards et al., 2006; van Kleunen et al., 2010). Furthermore, some invasive species may not outcompete native species but are exceptionally resistant to stress (Golivets & Wallin, 2018). Invasive species are also believed to be more responsive to environmental changes or disturbances in resources (light, nutrition, water, temperature, etc.), as they usually have higher ecological tolerance, which has been concluded to be the “fluctuating resources hypothesis (FRH)” (Davis et al., 2000; Davis & Pelsor, 2001; Pearson et al., 2018).

Temperature is a fundamental nonbiological factor in plant growth, production, and distribution, and the distribution of plants in terrestrial ecosystems is limited by heat shock and a cold damaging climate (Schulze et al., 2005). If the temperature rises during the winter, invasive plants may have a greater chance of overwintering and spreading to previously unsuitable areas (Hellmann et al., 2008; IPCC, 2018; Rahel & Olden, 2008). Compared with terrestrial ecosystems, aquatic systems have more thermal stability and a temperature buffering effect on environmental changes; however, they are nonetheless sensitive to climate change as marginal increases in temperature still have significant effects (Kraemer et al., 2017, 2021; Molles & Sher, 2019; Nickus et al., 2010; Tong et al., 2021; Woolway et al., 2021). Studies have shown that aquatic systems are vulnerable to invasion as climate change proceeds and warming has a negative impact on native aquatic species (Ma et al., 2010; Sorte et al., 2013; Thomaz et al., 2015). Free‐floating plants grow at the water–atmosphere interface and are sensitive to changes in adverse environmental factors; thus, these plants are frequently killed by low temperatures in winter. They may be more susceptible to climate change than the other three life‐forms of aquatic plants (emergent, floating‐leaved, and submerged plants) (May, 2007). However, a previous study has also shown that free‐floating plants are less affected than submerged plants in the response to eutrophication, which is also a part of global change, and their distribution range could expand (Zhang et al., 2017). However, there is relatively limited research concerning the relationship between climate change and the invasion of free‐floating plants and their overwintering functional trait performance variations in freshwater ecosystems.

Aquatic invasions have cost the world economy US$ 345 billion (Cuthbert et al., 2021). Among these, *Eichhornia crassipes* (water hyacinth) is the most prevalent and well‐known free‐floating invasive plant and is included in *100 of the World's Worst Invasive Alien Species* (Barrett, 2011; Hill et al., 2011; International Union for Conservation of Nature [IUCN], 2013). The plant was introduced into China as an ornamental plant and quickly escaped and thrived as a nuisance in China (Lolis et al., 2019; Wu & Ding, 2019). It can form dense, interlocking, and self‐stabilizing floating mats, inhibit sunlight, and block the exchange between water and air, thereby smothering submerged plants and benthos (Michelan et al., 2018; Scheffer et al., 2003; Yu et al., 2019). Under adverse environmental stress, for example, heavy metals, organic contaminants, and eutrophication, *E. crassipes* shows a higher tolerance than native plants (He et al., 2013; Mishra & Maiti, 2017; Villamagna & Murphy, 2010). Due to its high ecological tolerance and plasticity, it can successfully occupy a wide range of habitats under different climates (Chambers et al., 2008). However, as a tropical plant, low temperature is still a limiting factor determining its distribution (Pan et al., 2012). The Lake Taihu Basin, where we conducted this experiment is south of the Yangtze River, and is the northern overwintering boundary of *E. crassipes*, which suffers from freezing and dies in winter (Yu et al., 2019).

Originating from tropical South America, *E. crassipes* is a cold‐sensitive plant, and low temperatures severely limit its survival and growth (Hussner et al., 2021). This plant cannot survive at temperatures below 5°C and dies after several hours at temperatures below 0°C (Owens & Madsen, 1995). Previous studies have suggested that its distribution area may expand into cool waters with the help of global warming (Lu et al., 2007; Yang & Everitt, 2010). *Eichhornia crassipes* is not tolerant to cold conditions along its northern boundary, but under warming conditions, the plant can overwinter successfully in China; previous studies have shown that harsh winters strongly inhibit the survival, growth, reproduction, and distribution of this species (Liu et al., 2016; You et al., 2013, 2014; Yu et al., 2019). The plant can reproduce clonally, although it does form flowers in China (Wu & Ding, 2019; Yu et al., 2019). Fortunately, due to the lack of insects that pollinate this plant when flowering, it seldom produces fertile seeds for overwintering in China (Barrett, 1980; Gao et al., 2018). However, with the increase in the minimum temperature during the winter, *E. crassipes* may be able to overwinter successfully and spread quickly in spring. Due to warming conditions, *E. crassipes* can successfully overwinter and produce new ramets (asexual reproductive organisms) during the next growing season, which may aid the species in spreading further as global warming progresses (Liu et al., 2016). In China, field investigations have revealed that *E. crassipes* can overwinter by stranding and rooting in the littoral zones of several lakes during low‐flow periods, a novel behavior that has not been observed in its original American location (Venter et al., 2017; Wang et al., 2017; You et al., 2013). Therefore, in the invasive area of China, this makes the plant transition from a free‐floating plant life‐form to an emergent plant life‐form during the winter, transforming the plant from an annual to a perennial species (Fan et al., 2013; Penfound & Earle, 1948). Although this “floating‐emergent‐floating” life‐form conversion process of *E. crassipes* is unusual in aquatic plants, it is a novel biological invasion phenomenon (Wang et al., 2017; Yang & Everitt, 2010). It is indeterminate whether the transformation of the life‐form of *E. crassipes* can be viewed as a niche shift.

Several studies (Liu et al., 2016; You et al., 2013, 2014; Yu et al., 2019) have addressed the response of *E. crassipes* to low temperature or water drawdown during the winter. There is a lack of understanding of the mechanism and the possible ecological effects of this overwintering rooting behavior. In this study, we conducted a cross‐year mesocosm experiment during the winter under increasing temperature and water drawdown treatments. Plant functional traits, including growth, morphological, root topological, photosynthetic, and stoichiometric traits (Table 1), were recorded. We aimed to address the following questions: (1) Can specific plant functional traits underlie performance variations in the overwintering of *E. crassipes* in response to climate warming and water drawdown? (2) Will increasing temperature and water drawdown promote the overwintering of *E. crassipes* in the littoral zones of Lake Taihu Basin?

**TABLE 1:** Abbreviations and descriptions of the measured functional traits

	Abbreviations	Descriptions	Units
Growth traits	Total biomass	Total plant biomass	g
	RGR	Relative growth rate	mg g^−1^ d^−1^
Morphological traits	LA	Leaf area	cm^2^
	SLA	Specific leaf area	cm^2^ g^−1^
	RL	Root length	cm
	SRL	Specific root length	cm g^−1^
	Root *c*	Lateral root number *c*	
	Mean *a*	Mean rootlet number *a*	
Root topological indices	TI	Topological index TI	
	*q*_*a*_	Topological index *q*_*a*_	
	*q*_*b*_	Topological index *q*_*b*_	
Photosynthetic traits	*F*_v_ */F*_m_	Maximum quantum yield of photosystem II	
	*A*_a_	Net assimilation rate	μmol m^−2^ s^−1^
Stoichiometric traits	LNC	Leaf nitrogen concentration	mg g^−1^
	LPC	Leaf phosphorus concentration	mg g^−1^
	PNUE	Photosynthetic nitrogen‐use efficiency	μmol g^−1^ s^−1^
	PPUE	Photosynthetic phosphorous‐use efficiency	μmol g^−1^ s^−1^

## 2 MATERIALS AND METHODS

### 2.1 Study site

The mesocosm experiment was conducted at the Taihu Laboratory for Lake Ecosystem Research (TLLER), Dongshan branch (31.0331°N, 120.4217°E) in Suzhou city, Jiangsu Province, which is one of the national field stations of the Chinese National Ecosystem Research Network (CNERN). TLLER is also the sole member of the Global Lake Ecology Observation Network (GLEON) in China. Downstream of the Yangtze River Basin, Lake Taihu is the third largest lake in China, with an area of 2338 km^2^. It is a shallow lake with an average depth of 1.9 m. The mean annual precipitation in Lake Taihu Basin is 1172 mm (Taihu Basin Authority [TBA], 2020). With a subtropical climate, Suzhou's mean air temperature (AT) is 15.7°C; the hottest month is June with a mean AT of 28.2°C, and the coldest month is January with a mean AT of 3.0°C (http://js.weather.com.cn/jsqh/js13csqhtd/08/914714.shtml [*in Chinese*]). The lowest monthly mean AT in January was 0.7°C and the highest was 7.4°C during the 1981–2010 period (http://www.nmc.cn/publish/forecast/AJS/suzhou.html [*in Chinese*]). The Lake Taihu Basin is a sensitive area for biological invasion since it is located in one of the developed areas of the Yangtze River Delta in China. A previous study showed that *E. crassipes* has become the dominant problematic aquatic invasive species in the waters of the Lake Taihu Basin (Huang et al., 2020).

### 2.2 Experimental design

**FIGURE 1:**
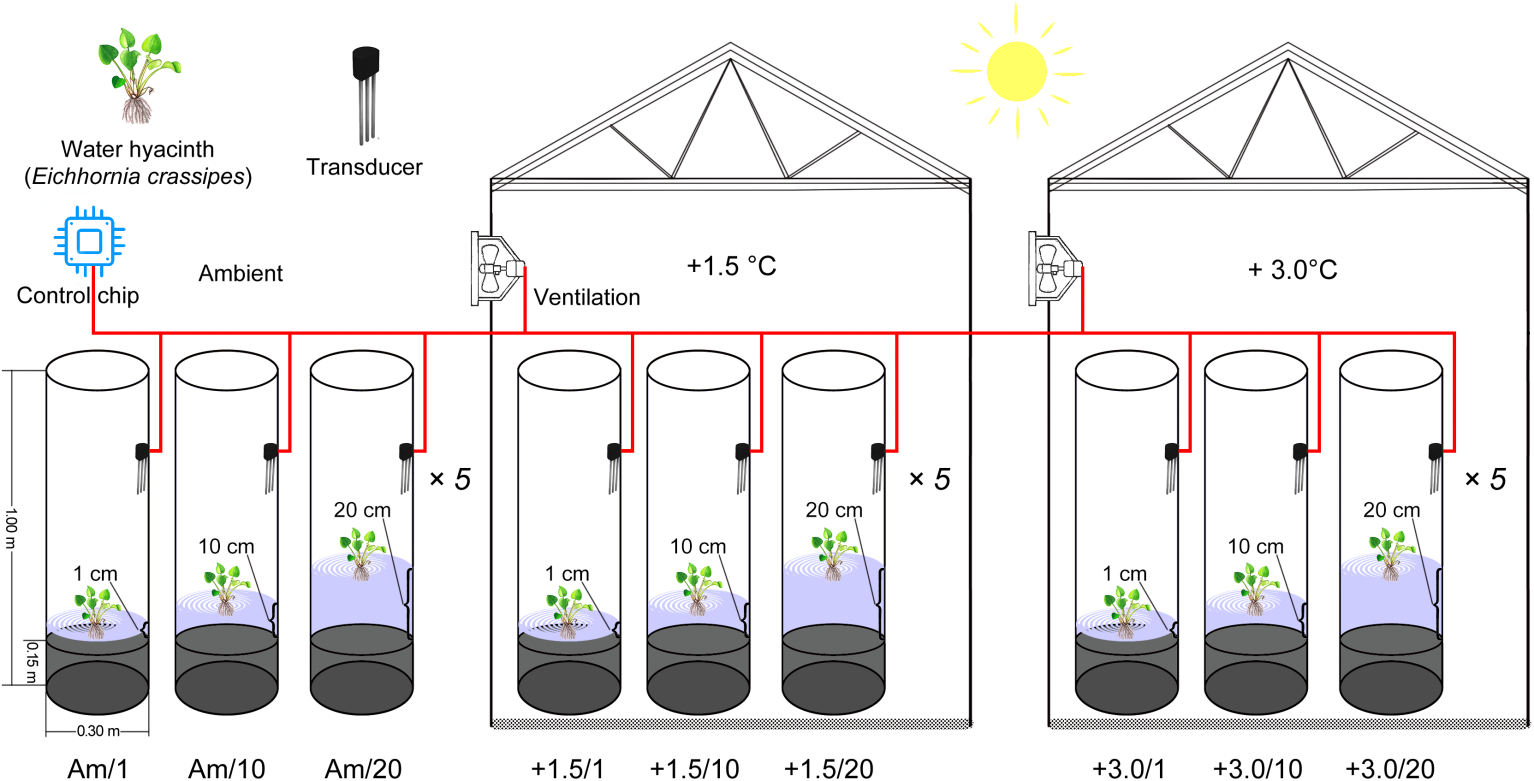
Design of the experiment. A factorial experiment was established and consisting of different air temperature (AT) treatments (ambient, +1.5°C, and + 3.0°C) combined with different water depths (WD) (1 cm, 10 cm, and 20 cm measured from the surface of the sediment) (abbreviated as Am/1, Am/10, Am/20, +1.5/1, +1.5/10, +1.5/20, +3.0/1, +3.0/10, and +3.0/20). The +1.5°C and 3.0°C treatments were performed in two conservatories with high‐power ventilation to increase air exchange between the inside and outside to reach the set air temperatures. The ventilation system was connected to YX‐WSD temperature and humidity transducers, and the transducers had sensitive water‐resistant probes that recorded the data. Each experimental setup had five replicates (*n* = 5).

On November 3rd, 2020, more than 200 young ramets of *E. crassipes* were collected from the littoral zone of Lake Taihu (31.0387°N, 120.4259°E) and cultivated in an outdoor pond (with a depth of 1.5 m and an area of appr. 400 m^2^) in the TLLER, Dongshan branch. A factorial experiment was established and consisted of different AT treatments (ambient, temperature rises of 1.5°C and 3.0°C) and combined with different levels of water drawdown (water depth [WD] of 1 cm, 10 cm, and 20 cm treatments measured from the surface of the sediment) (abbreviated as Am/1, Am/10, Am/20, +1.5/1, +1.5/10, +1.5/20, +3.0/1, +3.0/10 and +3.0/20) (Figure 1). The set temperature corresponded to the predicted temperature of the IPCC report, that is, global warming of 1.5°C (1.0°C to 1.8°C) under low GHG emissions and global warming of 3.0°C (2.1°C to 3.5°C) in the intermediate GHG emission scenarios (IPCC, 2018, 2021). The set water depths were based on our investigation in the winter of 2018–2019 in the littoral zones of Lake Taihu, namely, a value of 1 cm was used to imitate the stranding of *E. crassipes* on the moist littoral zone, a value of 10 cm was used to imitate the plant that could root in the littoral zone, and a value of 20 cm was used to imitate the plant floating on the water surface (Figure 1). No heating equipment was used; instead, we placed the +1.5°C and 3.0°C treatments in two conservatories with high‐power step‐less variable speed ventilation to promote air exchange between the inside and outside to obtain the set air temperature (Figure 1). The aim of this approach was to synchronize the temperature inside and outside the two conservatories. The whole system was programmed and controlled by an SY‐Temper‐4 temperature control chip (Shengyan Electronic Technology Co., Ltd), and ventilation was initiated automatically to maintain the two conservatories at the set temperature (Figure 1). The ventilation system was connected to YX‐WSD temperature and humidity transducers (Shengyan Electronic Technology Co., Ltd, Handan, Hebei, China), which possessed sensitive water‐resistant probes for data collection and were placed at a height of 75 cm in each cylinder (Figure 1). The air temperature inside and outside the two conservatories was recorded every 15 min.

The experiment was initialized on December 23rd, 2020, just after the winter solstice. Each group was replicated five times (*n* = 5), and a total of 45 (5 × 3 × 3) transparent polymeric methyl methacrylate (PMMA) cylinders (*d* = 0.30 m, *h* = 1.00 m) were used. The cylinders were placed inside (temperature rising of 1.5°C and 3.0°C treatments) and outside (ambient treatments) the two conservatories, and the ambient treatments were covered with caps when rain or snow was forecast (Figure 1). At the beginning of the experiment, 15 cm sediment (total nitrogen (TN) = 1.77 ± 0.13 mg g^−1^, total phosphorus (TP) = 0.32 ± 0.01 mg g^−1^, and total carbon (TC) = 6.47 ± 0.31 mg g^−1^, mean ± S.E.) from littoral zones of Lake Taihu were collected, air dried, sieved through a 15‐mesh sieve, and added to the cylinders. Water from Lake Taihu was poured into each cylinder to WDs of 1, 10, and 20 cm (measured from the surface of the sediment), and the water depths were maintained by adding lake water during the experimental period. Ramets with a height of 14 cm, 7 leaves, and 20–25 lateral roots were chosen, and one ramet was inserted into a cylinder. Fifteen ramets were selected and dried at 65°C for 72 h to measure the primary biomass (1.263 ± 0.031 g, mean ± SE). The experiment was harvested when the plants under the ambient treatments outside the two conservatories were in the senescence phase with their leaves drying and withering. The harvest occurred on January 23rd, 2021, which was a sunny day, and the experiment lasted for 31 days. Neither flowers nor asexual propagules of the plants formed during the experiment. Plant functional traits were measured afterward.

### 2.3 Microclimate

The lake water used for maintaining the water depth in the cylinders was measured every 3 days, and the water environmental parameters measured during the experiment were as follows: TN = 1.56 ± 0.26 mg g^−1^, TP = 0.10 ± 0.02 mg g^−1^, pH = 8.21 ± 1.27, conductivity = 359.8 ± 134.8 ms cm^−1^, salinity (SAL) = 0.21 ± 0.01‰, and dissolved oxygen (DO) = 6.09 ± 2.11 mg L^−1^ (mean ± SE). The water TN and TP were assessed using an NPW‐160 automatic total nitrogen and phosphorus COD analyzer (Hach Corp.). Other parameters were measured by a YSI Professional Plus water quality meter (YSI Inc.). The sunlight illuminance was recorded at 12:30 pm only on sunny days with an MQ‐510 underwater quantum flux sensor instrument (Apogee Electronics Corp.). The relative humidity was recorded with the mentioned YX‐WSD temperature and humidity transducers. Neither the sunlight illuminance nor the relative humidity significantly differed inside and outside the conservatories (Table 2).

**TABLE 2:** The sunlight illuminance and relative humidity during the experiment

	Sunlight illuminance (μmol m^−2^ s^−1^)	Relative humidity (%)
Ambient	943.7 ± 133.1a	47.05 ± 10.12a
+1.5°C	918.2 ± 189.0a	50.41 ± 9.18a
+3.0°C	922.3 ± 141.6a	49.23 ± 8.40a

*Note*: The data are presented as the mean ± SE. The same lowercase letters indicate that no significant differences were observed among the treatments (*p* < .05).

### 2.4 Plant growth and morphological traits determination

The plants were collected and washed gently with tap water, and then the roots, stems, and leaves were separated. The plant growth traits including the total biomass and relative growth rate (RGR), morphological traits leaf area (LA), specific leaf area (SLA), root length (RL), specific root length (SRL), lateral root number *c*, and mean rootlet number *a* were measured. The leaves and roots were scanned with an Expression 12000XL scanner (Seiko Epson Corp.). The LA was measured with a WinFolia leaf image analysis system (Regent Instruments Inc.). The RL was measured with a WinRHIZO root image analysis system (Regent Instruments Inc.). The leaves and roots were dried at 65°C for 72 h for the biomass measurement.

The RGR was calculated as follows:

$$\begin{array}{c} \mathrm{RGR}\;\left(\mathrm{mg}\;g^{-1}\;d^{-1}\right)=\left(\ln\;r_{2}-\ln\;r_{1}\right)/\left(t_{2}-t_{1}\right), \end{array}$$

where *r*_1_ is the initial dry plant biomass at initial time *t*_1_ and *r*_2_ is the dry plant biomass at harvest time *t*_2_. In this study, *t*_2_ − *t*_1_ = 31 days.

The plant SLA was calculated as follows:

$$\mathrm{SLA}\;\left({\mathrm{cm}}^{2}\;g^{-1}\right)=\mathrm{LA}/\text{leaf biomass}.$$

The SRL was calculated as follows:

$$\mathrm{SRL}\;\left(\mathrm{cm}\;g^{-1}\right)=\mathrm{RL}/\text{root biomass}.$$

### 2.5 Plant root topological traits determination

*Eichhornia crassipes* displays a distinctive poly‐herringbone branching, as its lateral root has only one main root and a considerable number of rootlets, which are herringbone‐like (Xie & Yu, 2003). Moreover, the secondary order of the whole plant is dichotomous (Huang et al., 2019). The root topological indices TI, *q*_*a*_, and *q*_*b*_ of *E. crassipes* were deduced according to the following methods (Huang et al., 2019):

$$\mathrm{TI}=\frac{\log\;a}{\log\;\mathrm{ca}};$$

$$q_{a}=\frac{a-1-\ln\;\mathrm{ca}/\ln\;2}{\mathrm{ca}-1-\ln\;\mathrm{ca}/\ln\;2};$$

$$q_{b}=\frac{\left(a+3-2/a\right)/2-1-\ln\;\mathrm{ca}/\ln\;2}{\left(\mathrm{ca}+1\right)/2-1/\mathrm{ca}-\ln\;\mathrm{ca}/\ln\;2}.$$

The TI, *q*_*a*_, and *q*_*b*_ of *E. crassipes* are indicated by the lateral root number *c* and the mean rootlet number *a*.

### 2.6 Plant photosynthetic traits determination

Three to five leaves of the plant in each cylinder were measured in situ with 4‐mm (0.13 cm^2^) diameter leaf clips after being dark‐incubated for 20 min before sunrise on the harvest day to ensure that all reaction centers in the chloroplasts were fully oxidized following the manual of the handy plant efficiency analyzer (PEA) chlorophyll fluorimeter (Hansatech Instruments Ltd). The leaf initial fluorescence *F*_0_ and maximum fluorescence *F*_m_ were measured, and the maximum quantum yield or energy trapping efficiency in photosystem II reaction centers, *F*_v_/*F*_m_, was calculated as follows:

$$F_{v}/F_{m}=\left(F_{m}-F_{0}\right)/F_{m}.$$

The plants were removed from each cylinder and measured in the morning on the harvest day in the field outside the two conservatories under ambient levels of O_2_ and CO_2_. Three leaves per treatment were randomly selected and measured with a 7 mm × 15 mm (1.75 cm^2^) PLC3 leaf cuvette, and they were stabilized in the cuvette for 10 min at 400 μmol mol^−1^ using a CO_2_ cartridge (ISI GmbH). The net assimilation rate *A*_a_ was measured by a CIRAS‐3 portable photosynthesis system (Portable Photosynthesis Systems).

### 2.7 Plant stoichiometric traits determination

Oven‐dried foliage was ground into a fine powder with a JXMF‐03 grinding mill (Shanghai Jingxin Industrial Development Co., Ltd.). The leaf nitrogen concentration (LNC) was determined by an EA3000 elemental analyzer (EuroVector). The leaf phosphorus concentration (LPC) was determined by a Prodigy inductively coupled plasma atomic emission spectrometer (ICP‐AES) (Teledyne Leeman Labs.). The foliar photosynthetic N‐use efficiency (PNUE) and photosynthetic P‐use efficiency (PPUE) were calculated as follows:

$$\text{PNUE}=\frac{A_{a}}{\mathrm{LNC}/\mathrm{SLA}};$$

$$\text{PPUE}=\frac{A_{a}}{\mathrm{LPC}/\mathrm{SLA}}$$

### 2.8 Data analysis

Prior to the data analysis, all data were verified and satisfied the assumptions of a normal distribution based on the Shapiro–Wilk test and homoscedasticity of variances based on Levene's test. A matrix consisting of AT and WD and all 17 functional traits was established to explore the effect of the two factors on the overwintering of *E. crassipes*. An unconstrained unimodal ordination model, that is, detrended correspondence analysis (DCA), was constructed in Canoco 5.0 (Microcomputer Power). The results revealed that the gradient lengths were shorter than 3, and therefore, it was preferential to use linear model principal components analysis (PCA) in this study. General linear models (GLMs) were applied with AT and WD as the primary factors to test their effects and interaction on the plant functional traits of *E. crassipes*, and post hoc pairwise comparisons of the means were performed to examine the differences between the treatments using Duncan's multiple range test at a significance threshold of *p* = .05. A one‐way ANOVA was applied to test the differences in the plant functional traits at a significance level of *p* = .05. The statistical analyses were conducted using SPSS Statistics 26 (IBM Corp.).

## 3 RESULTS

### 3.1 Temperature measurement

**FIGURE 2:**
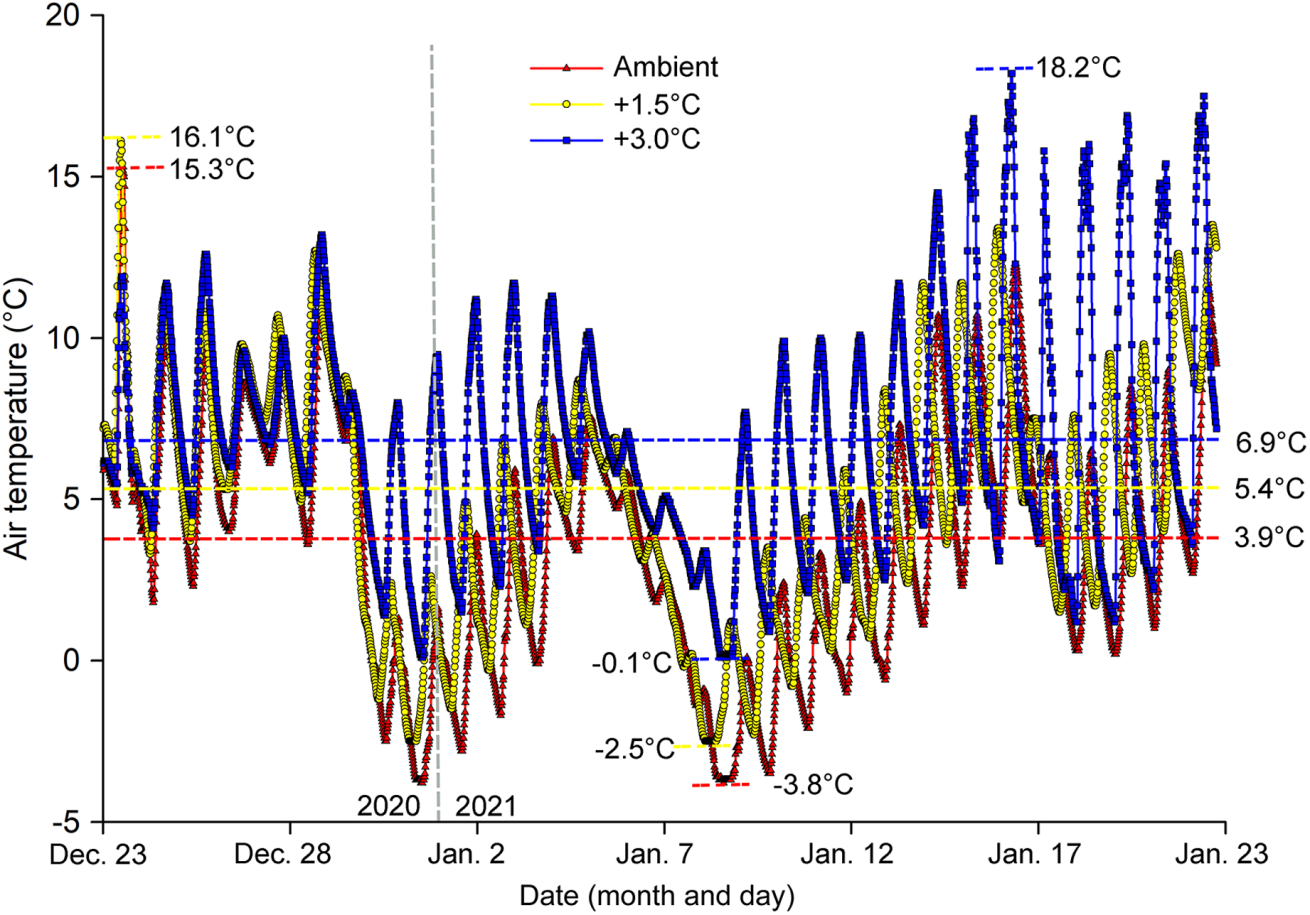
The air temperature under the ambient, +1.5°C, +3.0°C treatments from December 23rd, 2020, to January 23rd, 2021. The air temperatures were recorded every 15 min during the experimental period.

The mean ATs under the ambient, +1.5°C, and + 3.0°C treatments were 3.9°C, 5.4°C, and 6.9°C, respectively, during the experimental period (Figure 2). All three lowest temperatures −3.8°C (ambient), −2.5°C (+1.5°C treatment), and −0.1°C (+3.0°C treatment), were recorded on the night of January 8th, 2021 (snowfall occurred on January 7th) (Figure 2). The highest temperatures of 15.3°C (ambient), 16.1°C (+1.5°C treatment) and 18.2°C (+3.0°C treatment) were recorded on December 24th, 2020, December 24th, 2020, and January 16th, 2021, respectively (Figure 2).

### 3.2 The effect of warming and water drawdown on the functional traits of *Eichhornia crassipes*

**FIGURE 3:**
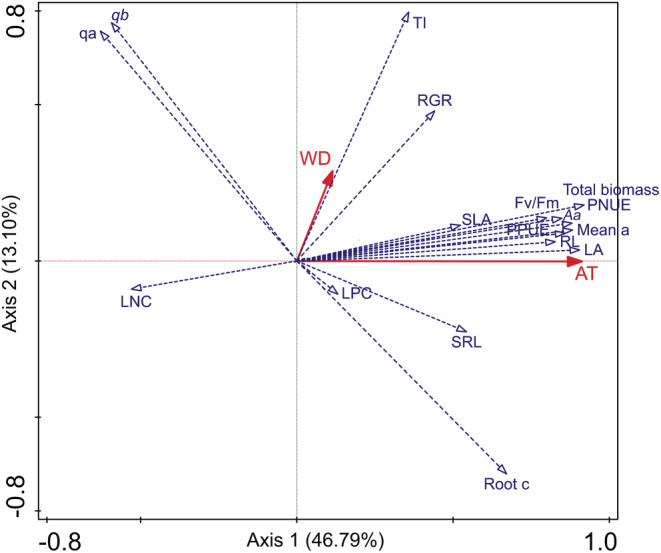
The ordination diagram of the principal component analysis (PCA) of air temperature (AT) and water depth (WD) along with the 17 functional traits. The definitions of the abbreviations and descriptions are shown in Table 1. Dashed blue vectors with open arrows represent 17 functional traits (response variables), and solid red vectors with filled arrows represent two explanatory variables, AT and WD. Arrows indicate the increase in values from the ordination center. The angle between the functional traits and factors represents their correlations, and the sharper the angle, the stronger the correlation.

The PCA results showed that the cumulative percentage variance of the relationship among AT and WD with 17 functional traits of the first two canonical axes was 59.89% (46.79% for axis 1 and 13.10% for axis 2) (Figure 3). AT had a closer relationship with these functional traits than WD (Figure 3).

AT and WD had a significant effect (*p* < .05) on all three growth traits, namely, the total biomass and RGR, in the experiment (Table 3). Except for RGR, the interaction effects between AT and WD were significant (*p* < .05) (Table 3).

**TABLE 3:** General linear models (GLMs) were applied with air temperature (AT) and water depth (WD) as the primary factors to test their effects and interaction on the plant functional traits of *E. crassipes* in the experiment.

	Air temperature (AT)		Water depth (WD)		AT × WD	
	*F*	*p*	*F*	*p*	*F*	*p*
Total biomass	241.297	**<.001**	33.866	**<.001**	9.018	**<.001**
RGR	209.838	**<.001**	19.693	**<.001**	1.536	.212
LA	119.196	**<.001**	20.428	**<.001**	3.269	**.022**
SLA	2.994	.063	3.808	**.032**	0.447	.774
RL	43.795	**<.001**	18.125	**<.001**	6.106	**.001**
SRL	3.721	**.034**	0.416	.663	0.108	.979
Root *c*	9.702	**<.001**	0.797	.458	0.150	.962
Mean *a*	48.187	**<.001**	13.676	**<.001**	2.656	.049
TI	4.880	**.013**	2.227	.123	0.440	.779
*q*_*a*_	9.864	**<.001**	0.557	.578	0.048	.995
*q*_*b*_	8.003	**.001**	0.412	.665	0.066	.992
*F*_v_ */F*_m_	48.401	**<.001**	19.921	**<.001**	2.758	**.042**
*A*_a_	26.122	**<.001**	3.628	**.037**	0.783	.543
LNC	8.300	**.001**	0.306	.738	1.057	.392
LPC	1.124	.336	2.515	.095	1.676	.177
PNUE	24.185	**<.001**	10.536	**<.001**	2.004	.115
PPUE	17.043	**<.001**	11.282	**<.001**	1.416	.249

*Note*: Significance at the *p* < .05 level is highlighted in bold.

AT had significant effects (*p* < .05) on the morphological traits LA, RL, SRL, root *c*, and mean *a*, but not on SLA, whereas WD had significant effects (*p* < .05) on LA, SLA, RL, and mean *a*, but not on SRL and root *c* (Table 3). There was a significant interaction effect between AT and WD on LA and RL (*p* < .05) but not on SLA, SRL, root *c*, or mean *a* (Table 3).

AT had significant effects (*p* < .05) on all three root topological indices TI, *q*_*a*_, and *q*_*b*_, while WD did not have significant effects (*p* < .05) on any of these traits. On all topological indices, the effects of the interaction between AT and WD had no significant effects (*p* < .05) (Table 3).

AT and WD both had significant effects (*p* < .05) on photosynthetic traits *F*_v_/*F*_m_ and *A*_a_ (Table 3). There was a significant interaction effect between AT and WD on *F*_v_/*F*_m_ (*p* < .05) but not on *A*_a_ (Table 3).

AT had significant effects (*p* < .05) on the stoichiometric traits LPC, PNUE, and PPUE but not LNC (Table 3). WD had significant effects (*p* < .05) on PNUE and PPUE but not on LNC or LPC (Table 3). The interaction effects of AT and WD were not significant on any of the four nutrient traits (*p* > .05) (Table 3).

### 3.3 Functional trait variations in *Eichhornia crassipes*

**FIGURE 4:**
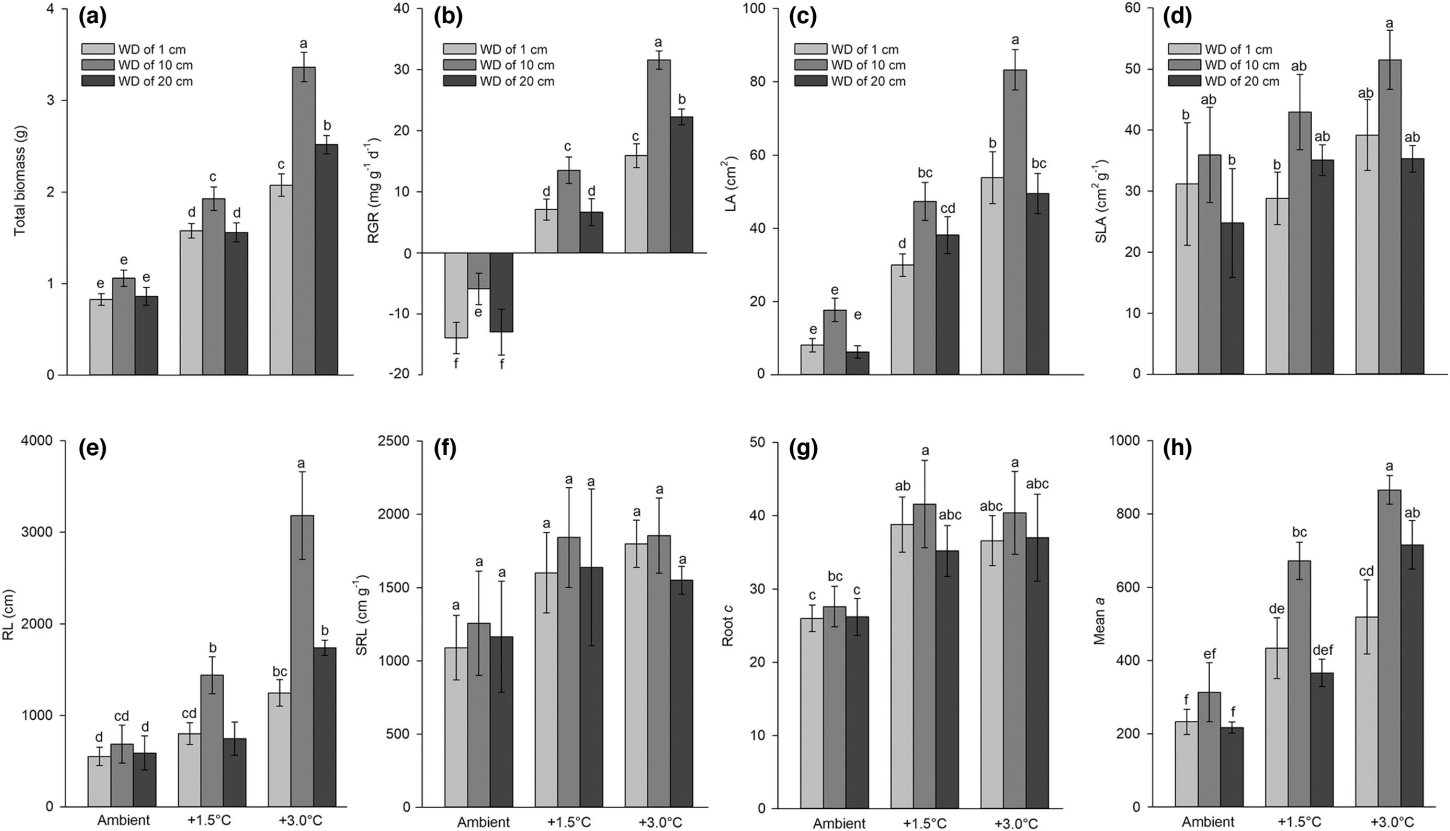
Plant growth traits (a) total biomass and (b) relative growth rate (RGR), and morphological traits (c) leaf area (LA), (d) specific leaf area (SLA), (e) root length (RL), (f) specific root length (SRL), (g) lateral root number *c* (root *c*), and (h) mean rootlet number *a* (mean *a*) under different air temperature (AT) and water depth (WD) treatments of *Eichhornia crassipes*. The values are the means (*n* = 5) ± SE. Different lowercase letters indicate significant differences among the treatments.

With increasing temperature, the total biomass increased, and the maximum biomass was observed under the +3.0/10 treatment, followed by the +3.0/20 treatment. The biomass did not significantly differ among the ambient treatments (Figure 4a). The RGRs in the ambient treatments were all below zero, indicating that low temperature strongly inhibited the growth of *E. crassipes* (Figure 4b). In contrast, the RGRs under the +1.5°C and +3.0°C treatments were higher than zero, indicating that warming strongly facilitated plant growth (Figure 4b).

With increasing temperature, the LA showed a sharp increase (Figure 4c). The +3.0/10 treatment exhibited the highest LA, followed by the +3.0/1, +3.0/20, and +1.5/10 treatments (Figure 4c). In contrast, the SLA showed a relatively stable fluctuation (Figure 4d). Regarding RL, the maximum value was 3183.90 ± 479.38 cm (mean ± SE) measured under the +3.0/10 treatment, and this value was significantly higher than those under the other treatments (Figure 4e), indicating that warming and water drawdown strongly facilitated the elongation of the roots of *E. crassipes*. However, similar to SLA, the SRL did not show significant differences, indicating that its roots maintained a stable fluctuation (Figure 4f). The mean rootlet number *a* showed a sharp increase (Figure 4h), whereas the lateral root number did not show a sharp increase under the warming and water drawdown treatments (Figure 4g). These results reveal that *E. crassipes* produces more rootlets and alters the length of roots to adjust to changes in environmental conditions.

**FIGURE 5:**
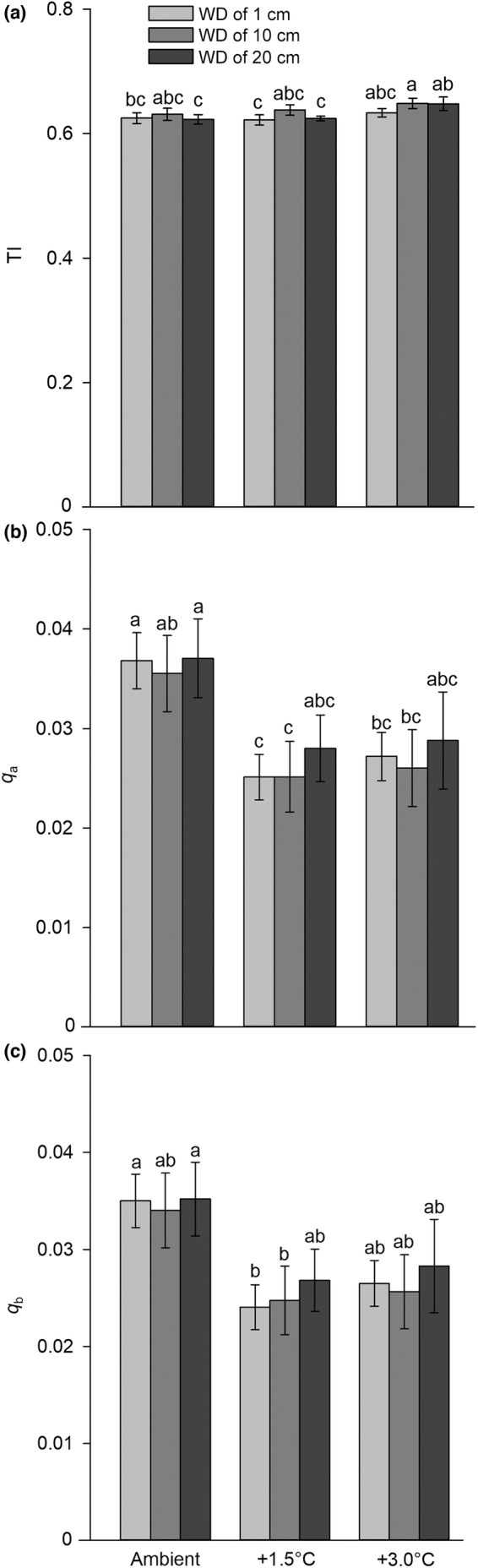
Root topological indices (a) TI, (b) *q*_*a*_, and (c) *q*_*b*_ under different air temperature (AT) and water depth (WD) treatments in *Eichhornia crassipes*. The values are the means (*n* = 5) ± SE. Different lowercase letters indicate significant differences among the treatments.

Among all treatments, the roots of *E. crassipes* under the +3.0/10 and +3.0/1 treatments exhibited the greatest topological index TI, followed by those under the 3.0/20 treatment (Figure 5a). The remaining treatments did not have a significant effect on TI, which showed a relatively stable fluctuation (Figure 5a). Regarding the topological indices *q*_*a*_ and *q*_*b*_, there were no significant treatment variations, indicating that the root topology did not change across the treatments (Figure 5b,c). The root structure of *E. crassipes* showed typical dichotomous branching, and root branching remained stable under the warming and water drawdown treatments (Figure 5b,c).

**FIGURE 6:**
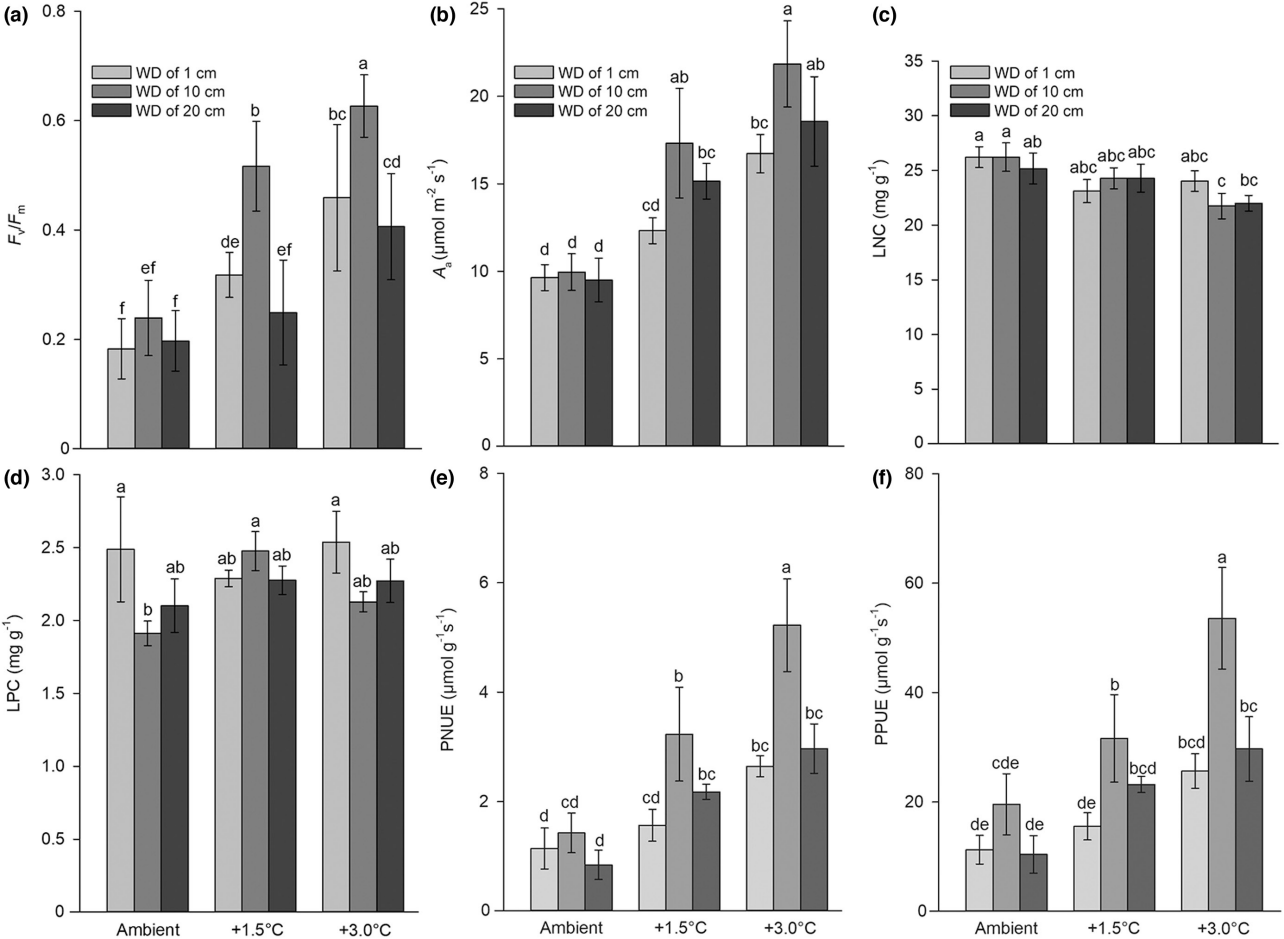
Plant photosynthetic traits (a) the maximum quantum yield of photosystem II *F*_v_ */F*_m_, (b) net assimilation rate *A*_a_, and plant stoichiometric traits (c) leaf nitrogen concentration (LNC), (d) leaf phosphorus concentration (LPC), (e) photosynthetic N‐use efficiency (PNUE), and (f) photosynthetic P‐use efficiency (PPUE) under different air temperature (AT) and water depth (WD) treatments of *Eichhornia crassipes*. The values are the means (*n* = 5) ± SE. Different lowercase letters indicate significant differences among the treatments.

The highest *F*_v_ */F*_m_ was measured under the +3.0/10 treatment, followed by the +1.5/10 and +3.0/1 treatments (Figure 6a). The highest net assimilation rate *A*_a_ was measured under the +3.0/10 treatment, followed by the +1.5/10 and +3.0/20 treatments (Figure 6b). The leaf nitrogen concentration (LNC) and leaf phosphorus concentration (LPC) showed a relatively stable trend among all treatments (Figure 6c,d). The PNUE and PPUE exhibited the same pattern among all treatments (Figure 6e,f). The highest PNUE and PPUE were both measured under the +3.0/10 treatments, followed by the +1.5/10 treatments (Figure 6e,f).

## 4 DISCUSSION

In the climate warming and water drawdown experiment, we discovered that the overwintering growth of the invasive plant *E. crassipes* was altered by both factors. Warming is a major factor determining the growth of the species. In general, climate tolerance impacts the geographic distribution of a species, particularly freeze tolerance, which is a strong limitation (Kerr & Kharouba, 2007). The influence of climate on plant traits also determines whether a species can thrive under specific environmental conditions (Chou et al., 2019; Dullinger et al., 2017; Gillard et al., 2017; Li et al., 2017). Plants are usually sessile, but free‐floating plants rely on wind, currents, and shipping to spread, which enhances their rapid invasion ability (Lacoul & Freedman, 2006). The drawback is that they usually dwell on the surface of water bodies, and freezing on the water surface is a substantial limitation of their survival during the winter (Chambers et al., 2008). The growth of *E. crassipes* is hampered by harsh winters, as the plant water content is 95%when *E. crassipes* is harvested (Pirie, 1960). Because of the damaging effect of the formation of ice crystals in plant cells, which causes them to rupture in addition to protein inactivation or denaturation (Hussner et al., 2021; Lambers & Oliveira, 2019). Our results show that the growth of *E. crassipes*, a tropical species, was hampered by low winter temperatures. However, controlling the temperature to within 1.5°C still facilitated the overwintering of *E. crassipes*. With continued global warming, the distribution of the plant may expand further north in China.

### 4.1 Climate warming and water drawdown facilitate overwintering growth of *Eichhornia crassipes*

The negative values of RGR and LA under the ambient treatments indicated that the harsh winter induced leaf fall of *E. crassipes*, which is consistent with a previous study showing that the leaves and stipes of the plant died and reduced the biomass and shoot height during the winter (You et al., 2013). However, the comparatively high RGR under the +3.0°C treatments demonstrated that the plant could grow rapidly under the appropriate conditions. According to previous studies, *E. crassipes* has one of the highest growth rates of any known plant (Lu et al., 2007; Pan et al., 2012). The plant can double its population in 11–18 days (Hill et al., 2011; May, 2007); consequently, the eradication of this fast‐growing plant during the summer may not be an ideal approach (Patel, 2012), and it may be better to eliminate rooted plants in the littoral zones in the water drawdown period during the winter.

Littoral zones have a higher temperature than open water, and the sediment is also richer in nutrients (Molles & Sher, 2019). *Eichhornia crassipes* is more competitive than native plants under favorable environmental conditions and exhibits higher plasticity than its congeneric species, especially at the root scale (Wang et al., 2017). The plant has a higher tolerance to frost if its roots are not affected (Pan et al., 2012). The considerable morphological plasticity of the laterally developed roots of *E. crassipes* may explain the high productivity and variations in the lateral roots (Xie & Yu, 2003). We found that the RL and LA, rather than SRL and SLA, showed an increasing tendency with increasing temperature and a proper water drawdown. A relatively low SLA indicates thicker, denser leaves, which increases the distance of water dispersion in the leaves and is beneficial for maintaining the water in the leaves and improving instantaneous water‐use efficiency (WUE) (Caplan & Yeakley, 2013; McDowell, 2002); similarly, a high SRL implies that plants can grow quickly because it is vital for nutrient and water foraging in terrestrial plants (Comas & Eissenstat, 2004; Tani et al., 2003). As water is relatively sufficient in freshwater ecosystems, the results may indicate that SLA and SRL may not be important for understanding plant responses in this study. The root structure is not constant, and plants can change their root branching in response to different environmental conditions (Beidler et al., 2015; Pagès, 2002; Šmilauerová & Šmilauer, 2002; Sorgonà & Cacco, 2002). We hypothesized that *E. crassipes* would increase its lateral root number *c* and mean rootlet number *a* or it might change its root branching under climate warming and water drawdown and may transform from dichotomous to herringbone branching, and vice versa. Compared to ambient conditions, the +1.5°C and +3.0°C treatments exhibited relatively low topological indices *q*_*a*_ and *q*_*b*_, which showed a greater dichotomous branching. However, the topological indices TI, *q*_*a*_, and *q*_*b*_ of the plant did not show significant differences among the different treatments, indicating that the plant conserved its root branching. *Eichhornia crassipes* has a unique root system in aquatic plants (Huang et al., 2019; Xie & Yu, 2003). The lateral roots of the plant had only one main root and a large number of rootlets (mean *a* in this study) (865.80 ± 38.88 under the +3.0/10 treatment), indicating typical herringbone branching. The plant may preserve its root branching, as revealed in this study, based on the two types of root branching (Huang et al., 2019), but whether the vernal water rise affects its root branching remains unknown.

The maximum quantum yield of photosystem II *F*_v_ */F*_m_ is an important index of the quantum efficiency of plant photosynthetic performance, and it is altered by biotic and abiotic stresses (Demiral & Türkan, 2006). The relatively low values under all experimental treatments indicated that the growth of *E. crassipes* was inhibited by water drawdown and low temperature during the winter. In addition, the rooted +3.0/10 treatment had the highest *F*_v_/*F*_m_ value, indicating that the increased temperature and rooting notably promoted the photosynthetic performance of photosystem II of *E. crassipes*, with better performance than that under the other treatments.

Foliar N is one of the most important resources, and most N is allocated to plant photosynthesis (Feng et al., 2009). Fan et al. (2013) found that *E. crassipes* has a relatively low LNC but achieved a higher PNUE in response to altered sediment nutrient levels. *Eichhornia crassipes* can redistribute its internal P to satisfy the requirements for growth (Wilson et al., 2005; Xie & Yu, 2003). A high PPUE in plants normally suggests a high rate of photosynthesis and high LPC, but it is also linked to short leaf life‐spans (Veneklaas et al., 2012; Wright et al., 2004, 2005). We noticed a similar tendency, as LNC and LPC maintained a relatively stable trend, but PNUE and PPUE increased with increasing temperature and proper water drawdown. When photosynthetic resource‐use efficiencies increase, the leaf nutrient content remains steady, indicating that the photosynthesis rate can be raised in response to changing temperature and water drawdown, which is beneficial for invasive plants to better adjust to environmental changes. In addition, the LNC and LPC under all treatments were higher than those in terrestrial plants (LNC = 18.6 ± 8.41 and LPC = 1.21 ± 0.99 mg g^−1^; means ± SD) in China (Han et al., 2005). However, both traits under all treatments were lower than those in a previous study showing the average LNC and LPC in free‐floating plants (LNC = 31.6 ± 2.36 and LPC = 3.70 ± 0.41 mg g^−1^; means ± SE) in China (Xia et al., 2014), which may indicate that the assimilation of the two nutrient elements are energetically costly and low temperatures inhibit their pathway.

### 4.2 Effort to control global warming at 1.5°C and its implication for controlling the spread of *Eichhornia crassipes*

Since 1960, China has experienced a 1.2°C increase in temperature (Piao et al., 2010). Controlling the temperature within 1.5°C is an ambitious goal for China (Duan et al., 2021; Gao et al., 2012). The FRH believes that an increase in the available resources will favor the invasibility of invasive species (Davis et al., 2000; Davis & Pelsor, 2001). Global warming can be defined as an increase in temperature, and our work provided direct evidence that an invasive plant could benefit from warming conditions, which is consistent with FRH. Invasive species may quickly acclimate to temperature increases, and in aquatic habitats, they are more susceptible to climate change than those in terrestrial ecosystems (Sorte et al., 2013). With global climate change, aquatic ecosystems will face a more severe threat of invasion. Rapidly changing climatic conditions favor opportunistic species (*r*‐strategy species) with strong dispersal ability (Malcolm et al., 2002). Despite the fact that it seems that no existing research has shown that *E. crassipes* is an *r*‐strategy species, the high plant growth rate, robust asexual production ability, and plentiful sexual production ability in its original area imply that the plant is an *r*‐strategy species.

Normally, invasive species, especially those originating from tropical zones, are believed to conserve their niche due to their lack of frost tolerance, and are unable to invade temperate regions (the “tropical niche conservatism (TNC) hypothesis,” also known as the “freezing tolerance hypothesis”) (Latham & Ricklefs, 1993; Liu et al., 2020; Petitpierre et al., 2012; Wiens & Donoghue, 2004). In addition to adjusting to the new environment, invasive species exhibit some behavioral changes (Fan et al., 2015; Nicotra et al., 2010; Webber et al., 2012; Xia et al., 2010). Meanwhile, they also alter their lifestyle to accommodate changes (Hulme, 2009). *Eichhornia crassipes* can currently establish roots in littoral zones of lakes, which may be viewed as a new invasion behavior (Wang et al., 2017; You et al., 2013; Yu et al., 2019). Water drawdown promoted the development of the root system and transformed the plant from a free‐floating life‐form to an emergent life‐form; this change can be considered a shift in niche and contradicts the TNC hypothesis. The new rooting behavior induced by water drawdown may be viewed as a unique growth adaptation strategy and a change in a niche that helps these plants invade empty niches left by dead free‐floating plants on the water surface caused by freezing during the winter. With continued global warming, the distribution of the plant may expand to northern China. Further studies of the response of both invasive and native species to climate warming and rising water in spring are still needed.

## AUTHOR CONTRIBUTIONS

**Xiaolong Huang:** Conceptualization (lead); writing – original draft (lead); writing – review and editing (lead). **Fan Ke:** Conceptualization (equal); writing – original draft (equal). **Qisheng Li:** Methodology (lead); writing – original draft (supporting). **Yu Zhao:** Investigation (equal); methodology (equal); project administration (supporting); writing – original draft (supporting). **Baohua Guan:** Conceptualization (supporting); writing – original draft (equal); writing – review and editing (equal). **Kuanyi Li:** Conceptualization (equal); funding acquisition (lead); supervision (lead); writing – original draft (equal); writing – review and editing (equal).

## CONFLICT OF INTEREST

There are no conflicts of interest to declare.

## Data Availability

Data are available at Dryad: https://doi.org/10.5061/dryad.c2fqz619p.
